# Ratiometric detection of pH fluctuation in mitochondria with a new fluorescein/cyanine hybrid sensor†
†Electronic supplementary information (ESI) available: Characterization of **Mito-pH**, emission spectra and photograph of **Mito-pH** solutions, linear fitting of the ratiometric response, and co-localization images at pH 8.50. See DOI: 10.1039/c4sc04021j



**DOI:** 10.1039/c4sc04021j

**Published:** 2015-03-16

**Authors:** Yuncong Chen, Chengcheng Zhu, Jiajie Cen, Yang Bai, Weijiang He, Zijian Guo

**Affiliations:** a State Key Laboratory of Coordination Chemistry , Coordination Chemistry Institute , School of Chemistry and Chemical Engineering , Nanjing University , Hankou Road No.22 , Nanjing 210093 , PR China . Email: heweij69@nju.edu.cn ; Email: zguo@nju.edu.cn ; Fax: +86-25-83314502 ; Tel: +86-25-83597066

## Abstract

The homeostasis of mitochondrial pH (pH_m_) is crucial in cell physiology. Developing small-molecular fluorescent sensors for the ratiometric detection of pH_m_ fluctuation is highly demanded yet challenging. A ratiometric pH sensor, **Mito-pH**, was constructed by integrating a pH-sensitive FITC fluorophore with a pH-insensitive hemicyanine group. The hemicyanine group also acts as the mitochondria targeting group due to its lipophilic cationic nature. Besides its ability to target mitochondria, this sensor provides two ratiometric pH sensing modes, the dual excitation/dual emission mode (D_ex_/D_em_) and dual excitation (D_ex_) mode, and its linear and reversible ratiometric response range from pH 6.15 to 8.38 makes this sensor suitable for the practical tracking of pH_m_ fluctuation in live cells. With this sensor, stimulated pH_m_ fluctuation has been successfully tracked in a ratiometric manner *via* both fluorescence imaging and flow cytometry.

## Abstract 2

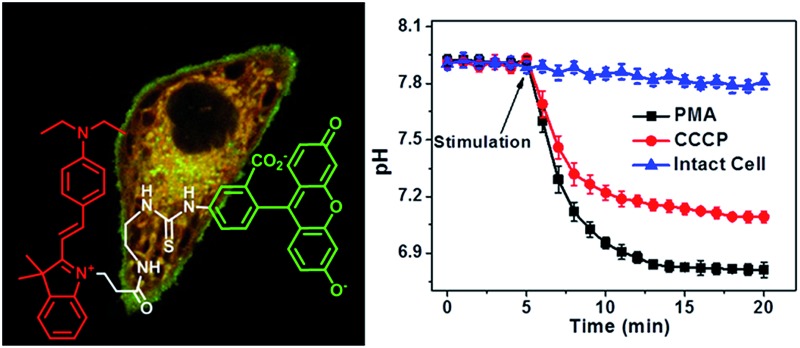
The first small-molecular ratiometric pH sensor with mitochondria targeting ability was constructed. With this sensor, the stimulated pH_m_ fluctuation in MCF-7 cells was monitored *via* both fluorescence confocal microscopy and flow cytometry.

## Introduction

Different from the acidic organelles, such as the lysosome and endosome, mitochondria display a slightly basic pH,^1^ and the proton gradient established across its inner membrane is essential to sustain the transmembrane potential for ATP production.^2^ Therefore, the development of facile and reliable methods to monitor the mitochondrial pH (pH_m_) in live cells is highly demanded in understanding the physiology and pathology of mitochondria. With the great success of intracellular pH (pH_i_) fluorescence imaging,^3–6^ developing a fluorescent pH sensor able to target mitochondria for pH_m_ imaging and flow cytometry should be a reliable approach to acquire the pH_m_ information *in situ*.^7^ Although mammalian pH_i_ ranges from slightly acidic in the lysosome and endosome (4.7–6.5) to slightly basic in active mitochondria (∼8.0), the physiological pH_m_ deviation is minor but of great significance for the understanding of mitochondria physiology. However, this minor pH_m_ deviation can be concealed in the case of using turn-on fluorescent sensors since the fluorescence intensity can be affected by local sensor concentration, microenvironment and imaging parameters, *etc.* Therefore, ratiometric pH sensors, which offer the possibility of self-calibration using dual emission, are more effective and advantageous in reducing the artefacts induced by the factors mentioned above.^8^ Although many ratiometric sensors for intracellular pH imaging have been reported,^5,6^ ratiometric sensors for pH_m_ are still rare and their design remains challenging. The nanoparticle-based design rationale for ratiometric pH_i_ sensors has been reported,^6^ yet they tend to localize in acidic endocytic compartments rather than the slightly basic mitochondria due to their uptake *via* endocytosis. Besides the successful FRET-based genetically encoded ratiometric pH sensors, which can be selectively expressed in mitochondria,^9^ small-molecular ratiometric pH_m_ sensors are especially appealing due to their simple staining procedure, easy reproducibility of results, and greater number of alternative emission wavelengths for imaging.^3^ Moreover, small-molecular sensors can be more readily endowed with pH sensitivity. Therefore, small-molecular sensors are among the most promising approaches to ratiometric pH_m_ imaging and tracking, and the sensing range from pH 6.50 to pH 8.20 is essential for practical pH_m_ tracking. Carboxy SNARF-1/AM, with no intrinsic ability to target mitochondria, has been reported as one of the very few small-molecular sensors for ratiometric pH_m_ imaging,^10^ its passive mitochondrial accumulation depending on both the higher efflux rate of the dye in the cytosol than in the mitochondria and the long incubation time for ester hydrolysis and efflux equilibrium. It is clear that a small-molecular ratiometric pH sensor with an intrinsic ability to target mitochondria, which has not been reported thus far, is very appealing.

**Scheme 1:**
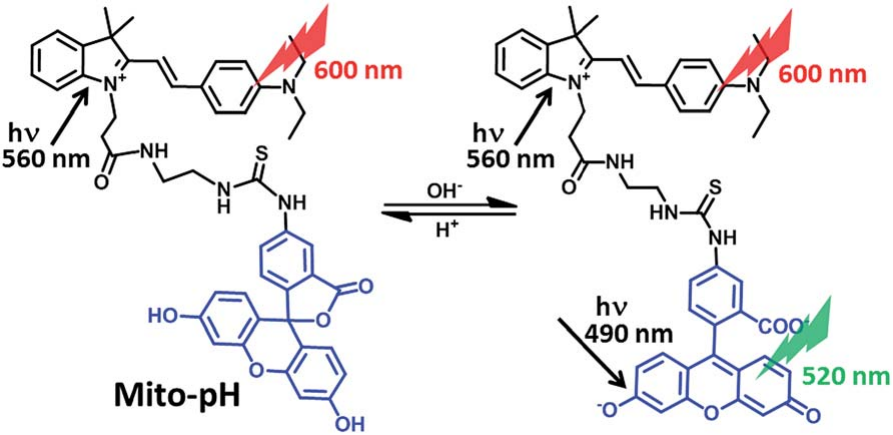
Chemical structure of **Mito-pH** and the proposed ratiometric pH sensing mechanism.

Herein, we report a small-molecular fluorescent sensor, **Mito-pH** (Scheme 1), for ratiometric pH_m_ imaging. The intrinsic ability of **Mito-pH** to target mitochondria was confirmed by a co-localization study, which originated from its lipophilic cationic cyanine moiety. It exhibits reversible pH sensing ability and a linear response range from pH 6.15 to 8.38. Besides ratiometric pH_m_ imaging *via* confocal microscopy, the general pH_m_ fluctuation upon stimulation has also been effectively monitored by flow cytometry in a ratiometric manner with this sensor.

## Results and discussion

### Design and synthesis of **Mito-pH**

**Mito-pH** was constructed *via* hybridizing a pH-sensitive fluorescein (spirolactone form) fluorophore with a pH-insensitive cyanine fluorophore (Scheme 1). Cyanines are well-known for their specific intracellular localization in mitochondria due to their lipophilic cationic nature,^11,12^ and the cyanine group was incorporated in this sensor as the reference fluorophore for ratiometric sensing as well as the mitochondria targeting group. It is proposed that the non-emissive spiro-xanthen-3-one turns into the emissive fluorescein at high pH, while the hemicyanine displays almost stable emission as the reference for ratiometric sensing.

**Scheme 2:**
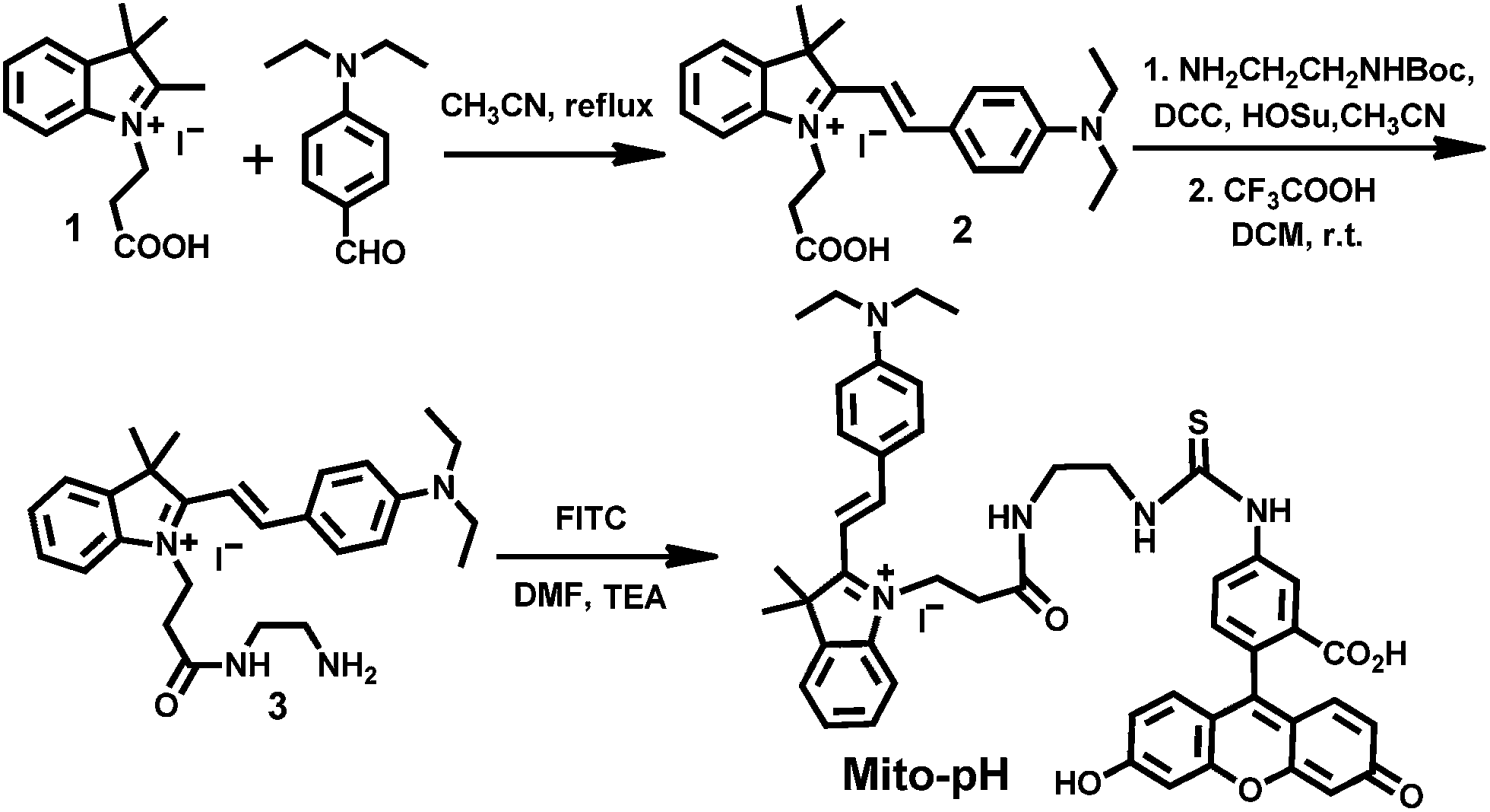
Synthesis of the compound **Mito-pH**.

To prepare this sensor, the cyanine derivative **2** was prepared in a reasonable yield *via* reacting 1-carboxylethyl-2-methylindolinum with *N*,*N*′-dimethylaminobenzaldehyde in reflux. The condensation of compound **2** with *N* ^1^-Boc-1,2-ethyldiamine followed by treatment with TFA resulted in a cyanine derivative with one amino tail (compound **3**). The condensation of compound **3** with fluorescein isothiocyanate (FITC) in the presence of triethylamine afforded the sensor **Mito-pH** (Scheme 2).

### Spectroscopic study and pH_app_-sensing behaviour of **Mito-pH**

**Fig. 1:**
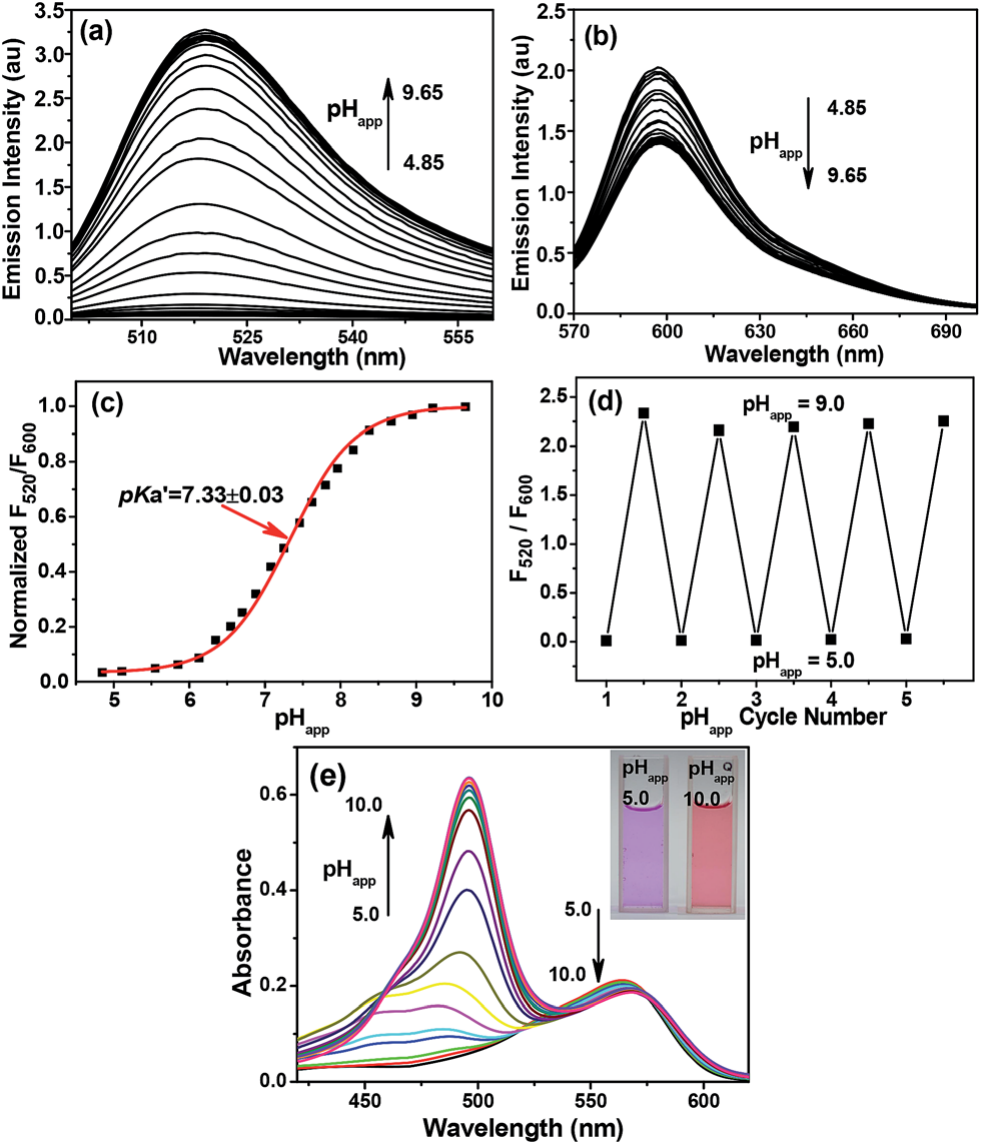
Emission spectra of 10 μM **Mito-pH** in DMSO–PBS buffer solutions (1 : 9, v/v) of different pH_app_ values determined upon excitation at (a) 490 nm and (b) 560 nm. (c) The related pH titration profile (■) based on the normalized emission ratio *F*_520_/*F*_600_ calculated from (a) and (b) and the fitting profile (red line). (d) Emission ratio *F*_520_/*F*_600_ of 10 μM **Mito-pH** in the same medium determined over consecutive pH_app_ cycles. (e) Absorption spectra of **Mito-pH** (10 μM) in the same media of different pH_app_. Inset: photograph of the solutions at different pH in ambient light.

Due to the precipitation of **Mito-pH** from the PBS buffer at acidic pH when the DMSO content is lower than 10%, a PBS buffer mixed with 10% DMSO was utilized as the medium to determine the emission spectra of **Mito-pH** at different apparent pH (pH_app_). As shown in Fig. 1, the emission spectra of **Mito-pH** upon excitation at 490 nm exhibit the characteristic emission band of FITC centered at 520 nm. A distinct enhancement of this band (>40-fold, Fig. 1a and S4a†) was observed upon increasing the pH_app_ of the medium from 4.85 to 9.65. The high pH_app_ induced fluorescein formation from spiro-xanthen-3-one is responsible for the enhancement. Its emission spectra upon excitation at 560 nm display the characteristic emission band of cyanine centered at 600 nm, which undergoes a decrement of ∼30% upon increasing the pH_app_ of the medium from 4.85 to 9.65 (Fig. 1b). The normalized ratio of emission at 520 nm (*λ*_ex_, 490 nm) to that at 600 nm (*λ*_ex_, 560 nm), *F*_520_/*F*_600_, increases from 0.023 to 0.99 upon increasing the pH_app_ of the medium from 4.85 to 9.65, and the quantum yield was enhanced from 2.6% to 25%.^13^ The apparent p*K*′_a_ was determined to be 7.33 ± 0.03 *via* fitting the pH titration profile based on the normalized *F*_520_/*F*_600_ (Fig. 1c).^14^ The linear range for the ratiometric response of **Mito-pH** is a pH_app_ of 6.15 to 8.38 (Fig. S5†), in which pH_m_ lies, supporting its ability to practically track pH_m_. This ratiometric pH-sensing behaviour originates from the different pH-sensing behaviours of the two hybridized fluorophores: FITC and the cyanine. In addition, the enhanced *F*_520_/*F*_600_ value of **Mito-pH** at pH_app_ 9.00 can be recovered to the original *F*_520_/*F*_600_ value at pH_app_ 5.00, and this reversible ratiometric pH sensing ability can be retained for at least 5 cycles in the pH_app_ range from 5.00 to 9.00 (Fig. 1d).

The absorption spectrum of **Mito-pH** at pH_app_ 9.00 shows a major absorption band centered at 490 nm which can be assigned as the FITC absorption band, and a minor band centered at 565 nm which can be assigned as the cyanine absorption band. However, the absorption spectrum at low pH_app_ (<5.00), shows only the cyanine band due to the absorption nature of the FITC spiro-form. The FITC band appears gradually and undergoes a significant enhancement with the pH_app_ of the medium increasing from 5.00 to 10.00, while the cyanine band remains almost stable. This ratiometric response can be clearly visualized from the distinct colour change of the **Mito-pH** solution from purple to pink during the titration process (Fig. 1e).

**Fig. 2:**
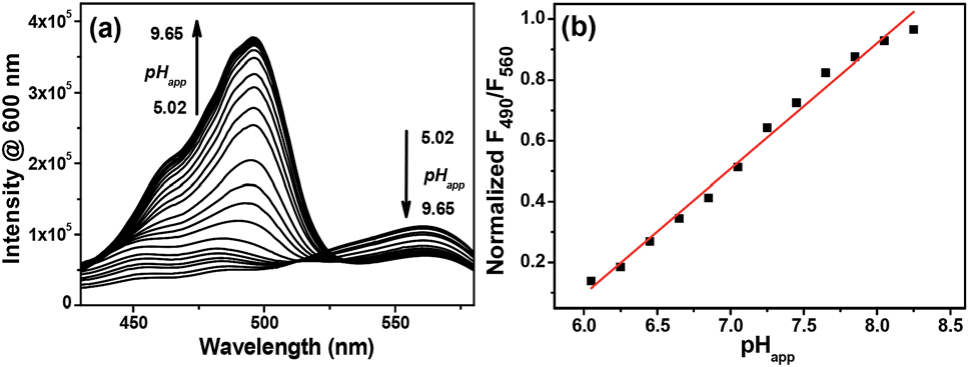
(a) Excitation spectra of 10 μM **Mito-pH** in DMSO–PBS buffer solutions (1 : 9, v/v) with pH ranging from 5.02 to 9.65; *λ*_em_: 600 nm. (b) Linear fitting (red line) of the related *F*_490_/*F*_560_ profile (■) of **Mito-pH**. *F*_490_/*F*_560_ is the normalized ratio of emission at 600 nm upon excitation at 490 nm to that upon excitation at 560 nm.

On the other hand, the excitation spectra of **Mito-pH** at different pH demonstrate that the excitation maximum at 560 nm at pH_app_ 5.02 can be shifted to 490 nm at pH_app_ 8.55. The ratio of emission at 600 nm upon excitation at 560 nm to that upon excitation at 490 nm, *F*_490_/*F*_560_, shows a linear enhancement with the pH_app_ of the medium increasing from 6.04 to 8.25 (Fig. 2). This additional dual excitation ratiometric pH sensing ability implies that **Mito-pH** is able to offer two ratiometric imaging modes: the dual excitation/dual emission (D_ex_/D_em_) mode and the dual excitation (D_ex_) mode. Therefore, this sensor possesses the advantage of the flexibility to match the laser/filter sets of microscopes and flow cytometers.

**Fig. 3:**
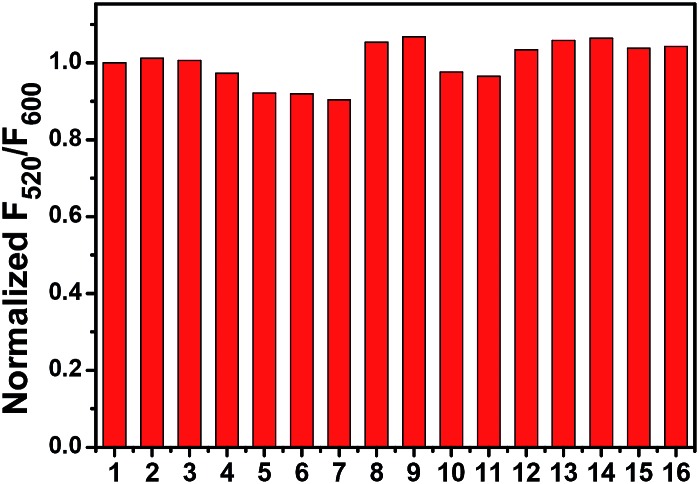
Normalized emission ratio *F*_520_/*F*_600_ of 10 μM **Mito-pH** in PBS solution (10 mM, pH 7.40, 10% DMSO–H_2_O, v/v) in the presence of different metal ions and biological redox species. 1: blank; 2: K^+^; 3: Na^+^; 4: Ca^2+^; 5: Mg^2+^ (2–5: 10 mM); 6: Zn^2+^; 7: Cu^2+^; 8: Fe^2+^; 9: Fe^3+^ (6–9: 10 μM); 10: GSH (10 mM); 11: Cys (1 mM); 12: H_2_O_2_; 13: ClO^–^; 14: NO; 15: O_2_^–^; 16: ˙OH (12–16: 100 μM). *F*_520_/*F*_600_ is the ratio of emission at 520 nm (*λ*_ex_, 490 nm) to that at 600 nm (*λ*_ex_, 560 nm).

The ratiometric fluorescent response of **Mito-pH** to different biological species was also investigated in PBS buffer (pH 7.40). The emission ratio *F*_520_/*F*_600_ exhibits a negligible change in the presence of essential metal ions (K^+^, Ca^2+^, Na^+^ and Mg^2+^ at 10 mM; Zn^2+^, Cu^2+^, Fe^2+^ and Fe^3+^ at 10 μM) and biologically related redox chemicals (GSH at 10 mM; Cys at 1 mM; H_2_O_2_, ClO^–^, NO, O_2_^–^ and ˙OH at 100 μM), demonstrating the specific ratiometric response of **Mito-pH** solely to pH_app_ (Fig. 3). All these suggest that **Mito-pH** might be a suitable candidate for ratiometric intracellular pH imaging.

### Ratiometric pH_m_ imaging behaviour of **Mito-pH**

**Fig. 4:**
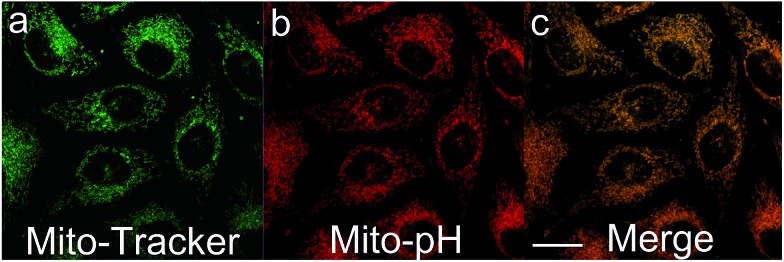
Pseudo-colour confocal fluorescence images of MCF-7 cells incubated firstly with DMEM containing **Mito-pH** (10 μM, 60 min) and Mito-Tracker Deep Red 633 (1 μM, 30 min) at 25 °C, followed by incubation with high K^+^ buffers (30 mM NaCl, 120 mM KCl, 1 mM CaCl_2_, 0.5 mM MgSO_4_, 1 mM NaH_2_PO_4_, 5 mM glucose, 20 mM HEPES, and 20 mM NaOAc) of pH 7.40 in the presence of 10.0 μM nigericin. (a) Fluorescence image obtained with the band path 660–750 nm upon excitation at 633 nm (Mito-Tracker channel); (b) fluorescence image obtained with the band path 560–640 nm upon excitation at 543 nm (**Mito-pH** channel); (c) overlay of (a) and (b). Scale bar: 20 μm.

The intrinsic ability of **Mito-pH** to target mitochondria was investigated in live MCF-7 cells at pH 7.40 and 8.50. The cells stained with **Mito-pH** (10 μM, 30 min, 25 °C) were co-stained further with the commercially available mitochondria dye Mito-Tracker Deep Red 633 (1 μM, 30 min) in the culture medium, and the pH_i_ was regulated by a following incubation with high K^+^ buffers of different pH containing nigericin (10 μM), an H^+^/K^+^ ionophore, to homogenize the intra- and extracellular pH. The imaging results show that the green image for the **Mito-pH** channel obtained upon excitation at 543 nm is almost identical to the red image for the Mito-Tracker channel obtained upon excitation at 633 nm (Fig. 4 and S6†). The overlay between the fluorescence images of **Mito-pH** and Mito-Tracker Deep Red 633 discloses a Pearson's correction coefficient of 0.96 at pH 7.40 and 0.93 at pH 8.50, suggesting the pH-independent ability of **Mito-pH** to target mitochondria. With this intrinsic ability to target mitochondria, the mitochondria staining equilibrium can be acquired much more quickly by **Mito-pH** than by carboxy SNARF-1/AM, which depends on the time-consuming dye ester hydrolysis and efflux process.

**Fig. 5:**
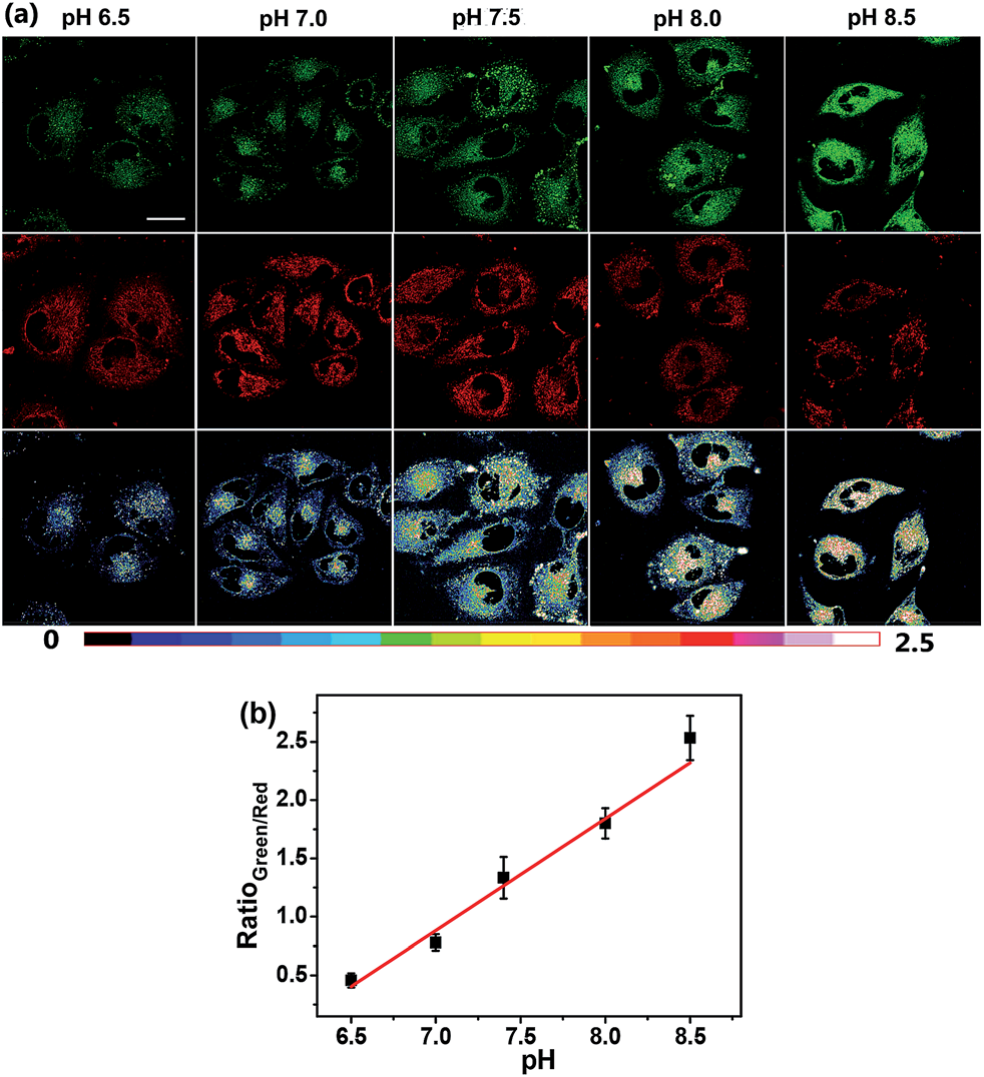
(a) Ratiometric imaging of MCF-7 cells stained by **Mito-pH** (10 μM in DEME with 0.1% DMSO, 30 min, 25 °C) upon further incubation with high K^+^ buffers (30 mM NaCl, 120 mM KCl, 1 mM CaCl_2_, 0.5 mM MgSO_4_, 1 mM NaH_2_PO_4_, 5 mM glucose, 20 mM HEPES, and 20 mM NaOAc) of different pH (6.50–8.50) in the presence of 10.0 μM nigericin. The green channel images (first row) were collected with a band path of 500–550 nm upon excitation at 488 nm; the red channel images (second row) were collected with a band path of 560–650 nm upon excitation at 543 nm. Pseudo-colour ratio images (third row) were obtained by mediating the green channel image with the red channel at the same pH. The colour strip is the ratio bar. Scale bar: 20 μm. (b) Calibration curve of pH_m_ based on the imaging results shown in (a).

With its intrinsic ability to target mitochondria confirmed, **Mito-pH** was further studied for its ratiometric pH_m_ imaging ability *via* a D_ex_/D_em_ mode. In this study, the intracellular pH calibration was carried out in MCF-7 cells using a standard procedure. MCF-7 cells were first incubated with the culture medium containing 10 μM **Mito-pH** for 30 min followed by a further incubation with high K^+^ buffers of different pH, also containing nigericin (10 μM). As shown in Fig. 5a, the green channel images (green channel: *λ*_ex_ 488 nm, band path 500–550 nm for FITC) display a gradually enhanced emission of fluorescein upon increasing the pH from 6.50 to 8.50, while the red channel images (red channel: *λ*_ex_ 543 nm, band path 560–650 nm for the hemicyanine) generally show a slightly decreased fluorescence of the hemicyanine. Moreover, the ratio images obtained *via* mediating the green with the related red channel images at the same pH by the program for ratiometric imaging show that the average ratio of green channel to red channel emission was enhanced linearly with the intracellular pH *i.e.* pH_m_ in this experiment (Fig. 5b). All these confirm the ratiometric pH_m_ imaging ability of **Mito-pH** *via* the D_ex_/D_em_ mode. With the ratiometric imaging calibration curve for pH_m_ (Fig. 5b), the ratiometric imaging of MCF-7 cells incubated with neutral PBS buffer without nigericin disclosed that the pH_m_ of intact cells is 7.9 ± 0.1, which is in agreement with the results determined using the genetically encoded fluorescent pH sensor.^4a,7^

**Fig. 6:**
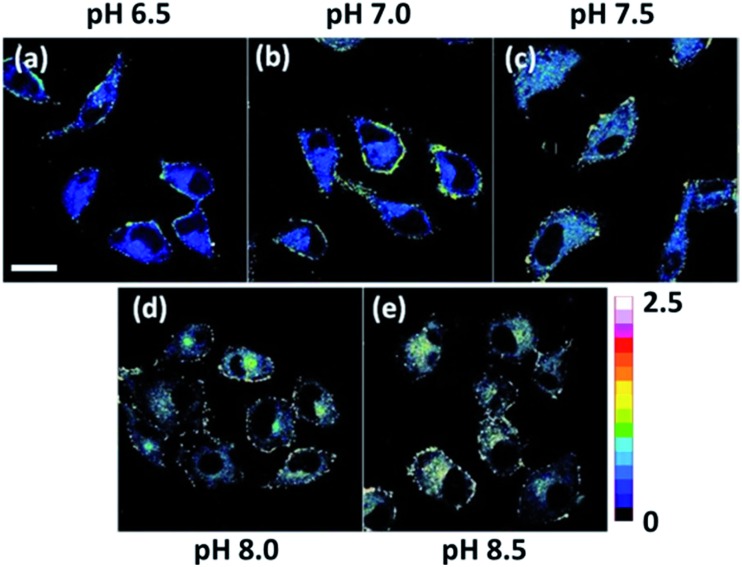
Pseudo-colour ratio images of MCF-7 cells stained by **Mito-pH** (10 μM in DEME with 0.1% DMSO, 30 min, 25 °C) upon further incubation with high K^+^ buffers (30 mM NaCl, 120 mM KCl, 1 mM CaCl_2_, 0.5 mM MgSO_4_, 1 mM NaH_2_PO_4_, 5 mM glucose, 20 mM HEPES, and 20 mM NaOAc) of different pH (6.50–8.50) in the presence of 10.0 μM nigericin. The imaging was carried out with a dual excitation mode (channel 1: *λ*_ex_: 488 nm; channel 2: *λ*_ex_: 543 nm; band paths for both channels are 560–650 nm) and the ratiometric images were obtained by mediating the channel 1 image with the related channel 2 image at the same pH. Scale bar: 20 μm.

As mentioned above, **Mito-pH** still displays a ratiometric sensing ability for pH *via* the D_ex_ mode. Therefore the ratiometric pH_m_ imaging ability was also investigated using the dual excitation imaging mode (channel 1: *λ*_ex1_: 488 nm; channel 2: *λ*_ex2_: 543 nm; band path 560–650 nm) in MCF-7 cells treated in the same way as shown for the D_ex_/D_em_ imaging mode. The ratiometric images were obtained *via* mediating the image obtained from channel 1 with the related channel 2 image at the same pH. These D_ex_ ratiometric images also display a linearly enhanced average ratio value inside the cells with the pH of the nigericin-containing high K^+^ buffer for cell incubation being raised from 6.50 to 8.50 (Fig. 6). This result also demonstrates the ratiometric pH_m_ imaging ability of **Mito-pH** *via* the D_ex_ imaging mode.

### Ratiometric flow cytometry for pH_m_ with **Mito-pH**

**Fig. 7:**
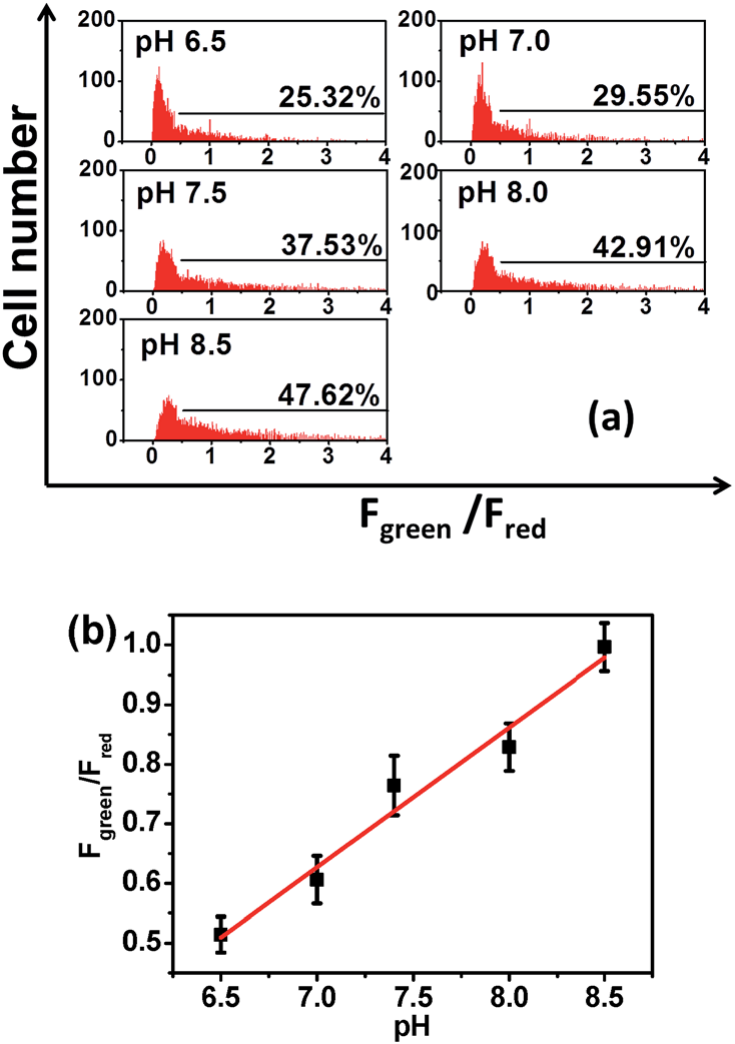
(a) pH_m_ flow cytometry of MCF-7 cells stained with **Mito-pH** upon incubation with high K^+^ buffers (30 mM NaCl, 120 mM KCl, 1 mM CaCl_2_, 0.5 mM MgSO_4_, 1 mM NaH_2_PO_4_, 5 mM glucose, 20 mM HEPES, and 20 mM NaOAc) of different pH values (6.50–8.50) in the presence of 10.0 μM nigericin. The *y*-axis is the cell number; the *x*-axis is the average *F*_green_/*F*_red_ ratio of the cells. The percentage shown for each tested pH value indicates the proportions of cells with *F*_green_/*F*_red_ higher than 0.5. Green channel: filter 530 ± 15 nm, *λ*_ex_: 488 nm; red channel: filter 610 ± 10 nm, *λ*_ex_: 561 nm. (b) Average *F*_green_/*F*_red_ ratio (■) of the MCF-7 cells stained with **Mito-pH** at different pH according to the data shown in (a) and its linear fitting (red line).

The tracking of pH_m_ in a large number of cells is crucial for understanding the general behavior of mitochondria, and the successful ratiometric pH_m_ imaging with **Mito-pH** inspires us to explore the possibility of ratiometric pH_m_ detection with this sensor *via* flow cytometry, which should offer a more reliable pH_m_ measurement due to the observation of a very large number of cells rather than the limited cell numbers in fluorescence imaging. The pH_m_ flow cytometry with this sensor was investigated in MCF-7 cells with a D_ex_/D_em_ mode. Therefore, the cells stained with **Mito-pH** were detected by recording the green channel emission (detection path 530 ± 15 nm, *λ*_ex_: 488 nm) and the red channel emission (detection path 610 ± 10 nm, *λ*_ex_: 561 nm). The cell distribution pattern was analysed *via* calculating the emission ratio of the green channel to the red channel (*F*_green_/*F*_red_). The results demonstrate that the number of cells with *F*_green_/*F*_red_ higher than 0.5 increases distinctly upon increasing the pH of the incubation medium from 6.50 to 8.50 (Fig. 7a). Moreover, the average *F*_green_/*F*_red_ ratio also displays a linear enhancement with the pH values (Fig. 7b). The similar ratiometric pH response of **Mito-pH** in flow cytometry and confocal imaging confirms the effectiveness of this sensor to monitor pH_m_ in live cells *via* the ratiometric manner.

### Tracking the stimulated pH_m_ fluctuation by **Mito-pH** *via* fluorescence imaging and flow cytometry

**Fig. 8:**
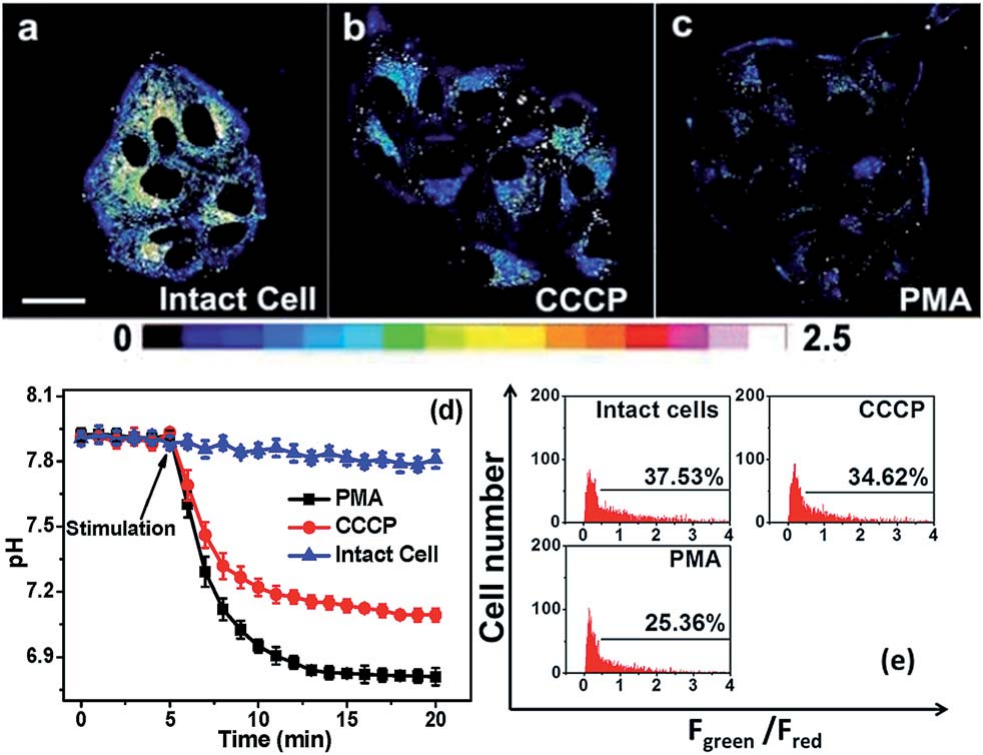
(a–c) Ratiometric images of MCF-7 cells loaded with **Mito-pH** in the presence of different stimulation agents. (a) Image of intact cells; (b) image of cells incubated with 10 μM CCCP; (c) image of cells incubated with 5 μg mL^–1^ PMA. Scale bar: 20 μm. The imaging conditions are the same as that shown in Fig. 5. (d) The calculated temporal pH_m_ profiles of the MCF-7 cells shown in (a–c) based on the calibration curve shown in Fig. 5b. (e) pH_m_ flow cytometry of MCF-7 cells stained with **Mito-pH** after 30 min of stimulation with 10 μM CCCP or 5 μg mL^–1^ PMA. The *y*-axis is the cell number; the *x*-axis is the average *F*_green_/*F*_red_ ratio. The percentage shown in each lane indicates the proportions of cells with a *F*_green_/*F*_red_ ratio higher than 0.5. Green channel: filter 530 ± 15 nm, *λ*_ex_: 488 nm; red channel: filter 610 ± 10 nm, *λ*_ex_: 561 nm.

The practical ratiometric pH_m_ imaging ability of **Mito-pH** was further applied in monitoring pH_m_ fluctuation upon different stimulation (Fig. 8). The MCF-7 cells stained with **Mito-pH** were incubated with carbonyl cyanide *m*-chlorophenylhydrazone (CCCP), which is a protonophore to uncouple the mitochondrial proton gradient across the inner membrane.^4*a*^ The ratiometric imaging *via* the D_ex_/D_em_ mode displays an instant and rapid drop in pH_m_ from 7.9 ± 0.1 to 7.2 ± 0.1 in the initial 5 min of CCCP incubation, and the pH_m_ tends toward stabilization at 7.1 ± 0.1 after 13 min of incubation (Fig. 8d). This result suggests that the CCCP-induced damage to oxidative phosphorylation might be correlated to the impairing of the mitochondria proton gradient. Since most of the intracellular oxidative stress occurs in mitochondria, exploring the influence of oxidative stress on pH_m_ should be helpful in understanding the role of pH_m_ in the electron transport chain and oxidative phosphorylation. Therefore, the pH_m_ fluctuation upon incubation with phorbol myristate acetate (PMA, 5 μg mL^–1^), which stimulates the generation of intracellular reactive oxygen species (ROS),^15^ was monitored *via* ratiometric imaging using **Mito-pH** as the imaging agent. The imaging demonstrates a rapid pH_m_ drop from 7.9 ± 0.1 to 6.8 ± 0.1 upon incubation with PMA. The temporal pH profile clearly indicates that the mitochondrial acidification stimulated by the PMA-induced oxidative stress can be finished within 5 min (Fig. 8d), and this acidification of the mitochondria might be attributed to the hydroxyl radicals triggered by PMA from the Fenton reaction between H_2_O_2_ and Fe^2+^.^16^

The pH_m_ deviation in MCF-7 cells upon stimulation with CCCP and PMA has also been determined *via* ratiometric flow cytometry with **Mito-pH**. The results demonstrate that the number of cells with *F*_green_/*F*_red_ higher than 0.5 decreases distinctly after 30 min of incubation in both cases. The average pH_m_ after incubation with CCCP is ∼7.20, while that after incubation with PMA is ∼6.60 according the calibration curve shown in Fig. 7b. This result implies that the improper reduction of O_2_ in the electron transport chain results in ROS formation to trigger the pH_m_ drop, disfavouring the oxidative phosphorylation.

## Conclusions

Hybridizing pH-sensitive FITC with a pH-insensitive cyanine led to the first small-molecular ratiometric pH sensor with an intrinsic ability to target mitochondria. The fluorescent ratiometric response achieved *via* the self-calibration with the cyanine reference leads to two different ratiometric pH imaging modes: the D_ex_/D_em_ mode and the D_ex_ mode, which offer more flexibility in pH_m_ imaging. The linear ratiometric pH response range from pH 6.15 to 8.38, the reversible pH sensing ability, the cell membrane permeability, and the mitochondria-targeting nature make this sensor especially suitable for the practical tracking of pH_m_ fluctuation in live cells *via* both ratiometric imaging and flow cytometry. Although the ratiometric imaging modes of **Mito-pH** are not as excellent as that of the single excitation mode in the tracking of very rapid physiological processes, the technological progress in confocal microscopy and flow cytometry can provide the D_ex_/D_em_ mode with a promoted scan rate and convenience similar to that of the single excitation mode, just as shown in the successful tracking of pH_m_ upon stimulation with PMA and CCCP. Moreover, this work provides not only a powerful imaging agent for pH_m_ but also an example of a fluorophore hybridizing strategy to construct ratiometric pH_m_ sensors, offering more accurate pH_m_ detection to clarify the physiological processes inside mitochondria.

## Experimental section

### Materials and general methods

All of the solvents used in sensor preparation were of analytic grade, while the solvents used in the spectroscopic study were of HPLC grade, and the purified water obtained from Millipore (>18.2 MΩ) was used for this study. The stock solutions of all the tested compounds were prepared from NaCl, KCl, MgCl_2_, CaCl_2_, ZnCl_2_, CuCl_2_, FeCl_2_, FeCl_3_, cysteine and glutathione using the purified water. ROS and RNS were prepared according to the reported procedures.^17^ The ^1^H NMR and ^13^C NMR spectra were recorded on a Bruker DRX-500 with TMS as the internal reference. High resolution mass spectrometric data were determined using an Agilent 6540Q-TOF HPLC-MS spectrometer. Fluorescence spectra were determined using a FluoroMax-4 spectrofluorometer with a 5 nm slit for both excitation and emission. Absorption spectra were recorded using a Shimadzu UV-3100 spectrophotometer. All pH measurements of media were accomplished using a Model PHS-3C meter.

### Confocal fluorescence imaging

MCF-7 cells were cultured in DMEM (Dulbecco's modified Eagle's medium) supplemented with 10% FBS (fetal bovine serum) in an atmosphere of 5% CO_2_ and 95% air at 37 °C. The ratiometric imaging of the MCF-7 cells was carried out by laser scanning confocal fluorescence microscopy (Zeiss LSM710).

For the mitochondria co-localization study, MCF-7 cells were stained firstly at 25 °C with the culture media (DMEM) containing 10 μM **Mito-pH** and 0.1% DMSO for 30 min, then 1 μM Mito-Tracker Deep Red 633 was added into the medium for an additional incubation (30 min). After removing the culture medium, the cells were further incubated with high K^+^ buffers (30 mM NaCl, 120 mM KCl, 1 mM CaCl_2_, 0.5 mM MgSO_4_, 1 mM NaH_2_PO_4_, 5 mM glucose, 20 mM HEPES, and 20 mM NaOAc) of pH 7.40 or 8.50 in the presence of 10.0 μM nigericin. The fluorescence images were obtained respectively with band path 560–640 nm upon excitation at 543 nm (**Mito-pH**) and band path 660–750 nm upon excitation at 633 nm (Mito-Tracker).

For the ratiometric pH_m_ calibration in live MCF-7 cells, the cells were stained with DMEM containing **Mito-pH** (10 μM) and 0.1% DMSO for 30 min at 25 °C, then the cells were further incubated (30 min) with high K^+^ buffers (30 mM NaCl, 120 mM KCl, 1 mM CaCl_2_, 0.5 mM MgSO_4_, 1 mM NaH_2_PO_4_, 5 mM glucose, 20 mM HEPES, and 20 mM NaOAc) of different pH values (6.50–8.50) in the presence of 10.0 μM nigericin. For the D_ex_/D_em_ ratiometric imaging mode, the green and red channel images were collected, with the band path of 500–550 nm upon excitation at 488 nm and the band path of 560–650 nm upon excitation at 543 nm, respectively. Pseudo-colour ratiometric images were obtained by mediating the green channel image with the red channel at the same pH. The pH_m_ calibration was finally obtained based on the average intracellular ratio values shown in the ratiometric images. For the D_ex_ ratiometric imaging mode, the fluorescence images from channels 1 and 2 were collected with a band path of 560–650 nm upon excitation at 488 (channel 1) and 543 (channel 2) nm, respectively, and the ratiometric images were obtained *via* mediating the channel 1 image with the related channel 2 image at the same pH.

For the ratiometric tracking of pH_m_ in MCF-7 cells upon stimulation with CCCP or PMA, the cells were stained with DMEM containing **Mito-pH** (10 μM) for 30 min at 25 °C. After incubation with **Mito-pH**, the cells were incubated respectively with CCCP (10 μM) and PMA (5 μg mL^–1^) for 15 min, and fluorescence images were obtained every 1 min for both the green and red channels with the D_ex_/D_em_ mode. The pH values at different times were calculated with the average ratio values obtained from the related ratiometric images according to the pH_m_ calibration shown in Fig. 5b.

### Ratiometric flow cytometric study

The flow cytometry tests were completed with a BD FACS AriaII using the D_ex_/D_em_ ratiometric mode (green channel: filter 530 ± 15 nm, *λ*_ex_: 488 nm; red channel: filter 610 ± 10 nm, *λ*_ex_: 561 nm). The MCF-7 cells were stained with the same method shown for ratiometric imaging.The stained MCF-7 cells were then incubated further with high K^+^ buffers (30 mM NaCl, 120 mM KCl, 1 mM CaCl_2_, 0.5 mM MgSO_4_, 1 mM NaH_2_PO_4_, 5 mM glucose, 20 mM HEPES, and 20 mM NaOAc) of different pH values (6.50–8.50) in the presence of 10.0 μM nigericin for an additional 30 min. After trypsinization with 0.25% pancreatin, all the cells were cooled with ice before the test. The fluorescence data were collected respectively from the green channel and red channels. The ratio of *F*_green_/*F*_red_ was calculated to give the pH_m_ calibration in the flow cytometric study. For the monitoring of the stimulated pH_m_ fluctuation, the fluorescence data were collected similarly after the MCF-7 cells stained with **Mito-pH** were treated with CCCP (10 μM) or PMA (5 μg mL^–1^) for 30 min. The pH_m_ was then obtained according to the detected average *F*_green_/*F*_red_ ratio based on the pH_m_ calibration for flow cytometry shown in Fig. 7b.

### Synthesis and characterization

#### Synthesis of **2**

Compound **1** (3.6 g, 10 mmol) and 4-(dimethylamino)benzaldehyde (1.8 g, 10 mmol) were dissolved in 20 mL CH_3_CN, and the reaction mixture was refluxed with stirring for 12 h and then evaporated *in vacuo*. The residue was purified by column chromatography on silica gel (CH_2_Cl_2_/MeOH, 20 : 1 v/v) to give **2** (3.0 g) as a dark purple solid. Yield: 59%. ^1^H NMR (500 MHz, CD_3_OD, ppm): *δ* 1.30 (t, *J* = 7.5 Hz, 6H), 1.82 (s, 6H), 2.96 (t, *J* = 7.5 Hz, 2H), 3.64 (q, *J* = 6.7 Hz, 4H), 4.76 (t, *J* = 7.5 Hz, 2H), 6.93 (d, *J* = 10.0 Hz, 2H), 7.30 (d, *J* = 15 Hz, 1H), 7.49 (t, *J* = 7.5 Hz, 1H), 7.56 (t, *J* = 10.0 Hz, 1H), 7.64 (m, *J* = 8.8 Hz, 2H), 7.97 (d, 10.0 Hz, 2H), 8.33 (d, *J* = 15.0 Hz, 1H). ESI-HRMS (*m*/*z*, positive mode): calcd 391.2386, found 391.2381 for [M – I]^+^.

#### Synthesis of **3**

Compound **2** (500 mg, 1 mmol), DCC (250 mg, 1.2 mmol), and HOSu (140 mg, 1.2 mmol) were mixed in 25 mL CH_3_CN, and then *tert*-butyl(2-aminoethyl)carbamate (160 mg, 1 mmol) was added into the mixture. The solution was stirred at room temperature for 6 hours. After removing the solvent *in vacuo*, the residue was purified by column chromatography on silica gel (CH_2_Cl_2_/MeOH, 10 : 1 v/v) to give a purple solid. The resulting solid (330 mg, 0.5 mmol) was dissolved in 5 mL CH_2_Cl_2_, then 3 mL of TFA was added dropwise into the solution. The mixture was stirred at room temperature for 1 hour. The solvent and TFA were removed *in vacuo*, and the resulting residue was purified by column chromatography on silica gel (CH_2_Cl_2_/MeOH, 9 : 1 v/v) to give **3** (250 mg). Yield: 54%. ^1^H NMR (500 MHz, MeOD, ppm): *δ* 1.30 (t, *J* = 7.5 Hz, 6H), 1.83 (s, 6H), 2.06 (s, 2H), 2.97 (t, *J* = 7.5 Hz, 2H), 3.64 (q, *J* = 8.3 Hz, 4H), 4.76 (t, *J* = 7.5 Hz, 2H), 6.93 (d, *J* = 10.0 Hz, 2H), 7.31 (d, *J* = 15.0 Hz, 1H), 7.49 (t, *J* = 7.5 Hz, 1H), 7.56 (t, *J* = 7.5 Hz, 1H), 7.65 (q, *J* = 8.3 Hz, 2H), 7.96 (d, *J* = 5.0, 2H), 8.33 (d, *J* = 15.0 Hz, 1H). ^13^C NMR (126 MHz, CD_3_OD, ppm): *δ* 154.84, 128.90, 127.49, 122.56, 113.29, 112.48, 103.98, 44.97, 41.93, 40.10, 36.67, 33.67, 26.50, 11.95 ppm. ESI-HRMS (*m*/*z*, positive mode): calcd 433.2967, found 433.2971 for [M – I]^+^.

#### Synthesis of **Mito-pH**

Compound **3** (560 mg, 1.0 mmol), FITC (389 mg, 1.0 mmol), and triethylamine (1 mL) were dissolved in 15 mL DMF, and the mixture was stirred at room temperature under N_2_ for 4 hours. The solvent was removed *in vacuo*, and the resulting residue was purified by column chromatography on silica gel (CH_2_Cl_2_/MeOH, 8 : 1 v/v) to give **Mito-pH** (120 mg). Yield: 13%. ^1^H NMR (500 MHz, *d*-DMSO, ppm): *δ* 1.16 (t, *J* = 7.5 Hz, 6H), 1.76 (s, 6H), 2.73 (t, *J* = 5.0 Hz, 2H), 3.19 (d, *J* = 10.0 Hz, 2H), 3.54 (q, *J* = 6.7 Hz, 6H), 4.77 (t, *J* = 5.0, 2H), 6.58 (q, *J* = 6.7 Hz, 4H), 6.70 (s, 2H), 6.87 (d, *J* = 10.0 Hz, 2H), 7.16 (d, *J* = 5.0 Hz, 2H), 7.30 (d, *J* = 15.0 Hz, 1H), 7.45 (t, *J* = 7.5 Hz, 1H), 7.53 (t, *J* = 7.5 Hz, 1H), 7.69 (d, *J* = 10.0 Hz, 1H), 7.77 (t, *J* = 10.0 Hz, 2H), 8.07 (d, *J* = 10.0 Hz, 2H), 8.32 (t, *J* = 7.5, 2H), 8.40 (s, 1H), 8.54 (s, 1H). ^13^C NMR (126 MHz, *d*-DMSO, ppm): *δ* 13.01, 27.03, 34.21, 38.43, 42.48, 43.10, 44.92, 51.04, 102.73, 105.05, 110.19, 112.52, 113.08, 113.88, 116.34, 122.63, 123.14, 124.42, 126.99, 127.67, 129.13, 129.42, 131.98, 137.33, 141.49, 142.05, 142.99, 147.29, 149.54, 152.37, 153.16, 154.80, 160.05, 169.07, 169.86, 179.93, 181.18. ESI-HRMS (*m*/*z*, positive mode): calcd 822.3325, found 822.3322 for [M – I]^+^.
