# Supplementary material for: Ratiometric detection of pH fluctuation in mitochondria with a new fluorescein/cyanine hybrid sensor
Source: Chem Sci. 2015 Mar 16;6(5):3187–94. doi: 10.1039/c4sc04021j (PMC5490428; doi:10.1039/c4sc04021j)
Supplement: Supplementary file 1 [file SC-006-C4SC04021J-s001.pdf]

Electronic Supplementary Material (ESI) for Chemical Science.
This journal is © The Royal Society of Chemistry 2015

Supporting Information

*for*

# Ratiometric detection of pH fluctuation in mitochondria with a new fluorescein/cyanine hybrid sensor

Yuncong Chen, Chengcheng Zhu, Jiajie Cen, Yang Bai, Weijiang He,* and Zijian Guo*

State Key Laboratory of Coordination Chemistry, Coordination Chemistry Institute, School of Chemistry and Chemical Engineering, Nanjing University, Hankou Road No.22, Nanjing 210093, PR China

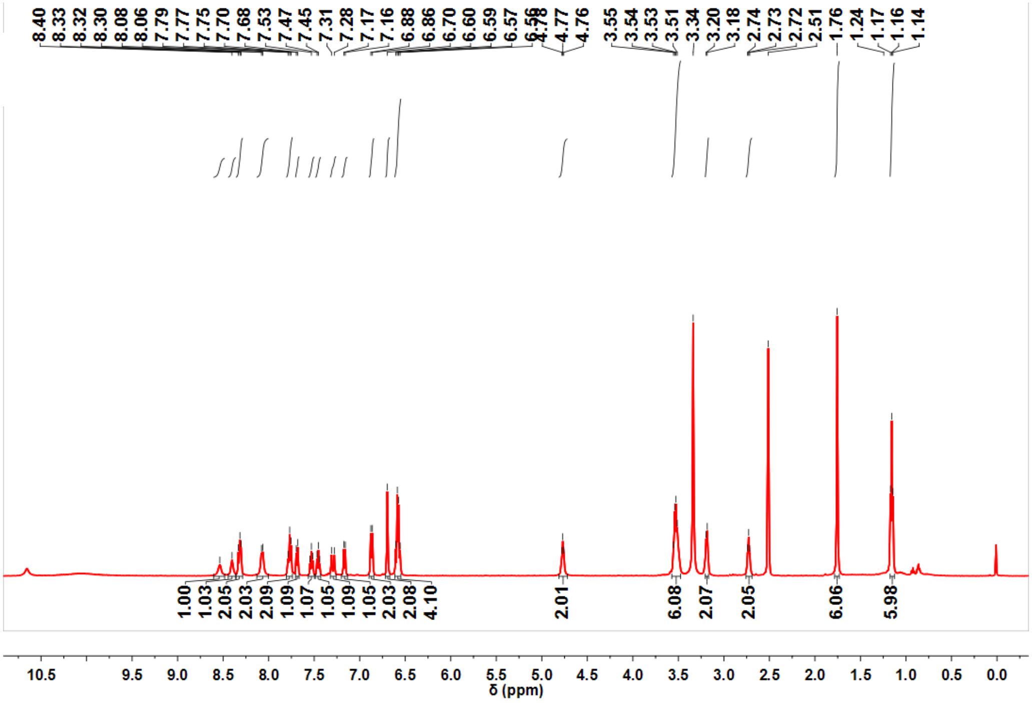


**Fig. S1** $^{1}H$ NMR spectrum of **Mito-pH** in DMSO-$d_6$.

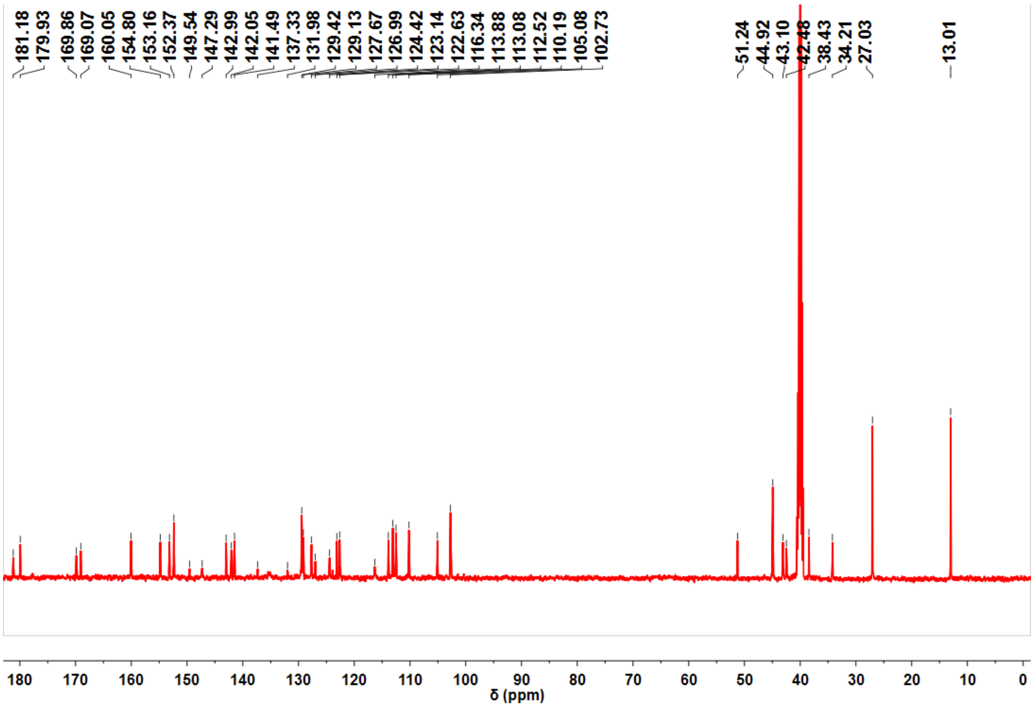


**Fig. S2**. $^{13}C$ NMR spectrum of **Mito-pH** in DMSO-$d_6$.

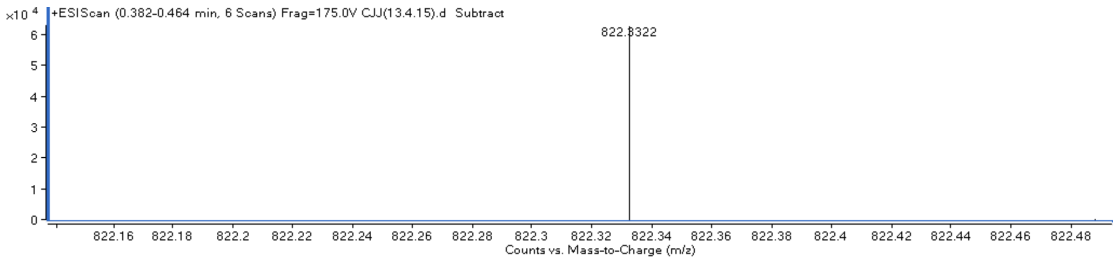


**Fig. S3** ESI-HRMS of **Mito-pH**.

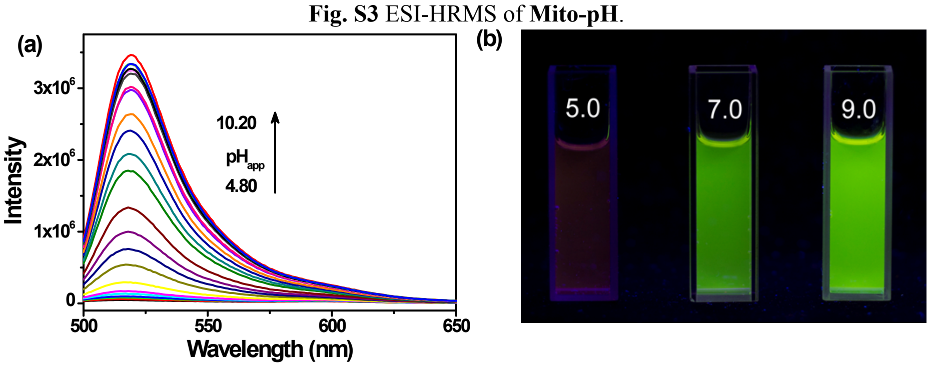


**Fig. S4** (a) Emission spectra of 10 μM **Mito-pH** in PBS buffer of different pH values containing 10% DMSO(v/v). $\lambda_{ex}$, 490 nm. (b) Photograph of the fluorescence of **Mito-pH** in this media of different pH values upon irradiation with a UV-lamp of 365 nm.

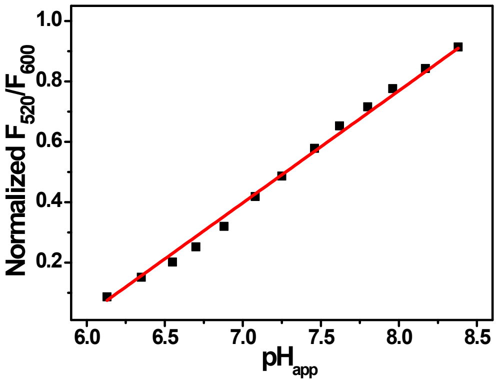


**Fig. S5** Emission ratio $F_{520}/F_{600}$ (o) of **Mito-pH** (10 μM) in PBS buffer of different $pH_{app}$ values containing 10% DMSO(v/v) and its linear fitting (red line). $F_{520}/F_{600}$ is the normalized ratio of emission at 520 nm ($\lambda_{ex}$, 490 nm) to that at 600 nm ($\lambda_{ex}$, 560 nm).

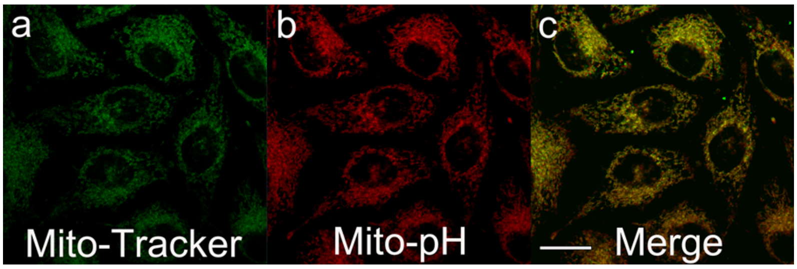


**Fig. S6** Pseudo-colour confocal fluorescence images of MCF-7 cells incubated firstly with DMEM containing **Mito-pH** (10 μM, 60 min) and Mito-Tracker Deep Red 633 (1 μM, 30 min) at 25°C, followed by incubation with high $K^+$ buffers (30 mM NaCl, 120 mM KCl, 1 mM $CaCl_2$, 0.5 mM $MgSO_4$, 1 mM $NaH_2PO_4$, 5 mM glucose, 20 mM HEPES, and 20 mM NaOAc) of pH 8.50 in the presence of 10.0 μM nigericin. (a) Fluorescence image obtained with the band path 660-750 nm upon excitation at 633 nm (Mito-Tracker channel); (b) fluorescence image obtained with the band path 560-640 nm upon excitation at 543 nm (**Mito-pH** channel); (c) overlay of (a) and (b). Scale bar: 20 μm.
